# A cortical field theory – dynamics and symmetries

**DOI:** 10.1007/s10827-024-00878-y

**Published:** 2024-10-01

**Authors:** Gerald K. Cooray, Vernon Cooray, Karl Friston

**Affiliations:** 1 Karolinska Institutet, Stockholm, Sweden; 2 Department of Electrical Engineering, Uppsala University, Uppsala, Sweden; 3 The Wellcome Centre for Human Neuroimaging, Queen Square Institute of Neurology, University College London, London, UK

**Keywords:** Neural fields, Lagrangian dynamics, Epileptic seizure generation, Pattern formation, Spectral power distribution

## Abstract

We characterise cortical dynamics using partial differential equations (PDEs), analysing various connectivity patterns within the cortical sheet. This exploration yields diverse dynamics, encompassing wave equations and limit cycle activity. We presume balanced equations between excitatory and inhibitory neuronal units, reflecting the ubiquitous oscillatory patterns observed in electrophysiological measurements. Our derived dynamics comprise lowest-order wave equations (i.e., the Klein-Gordon model), limit cycle waves, higher-order PDE formulations, and transitions between limit cycles and near-zero states. Furthermore, we delve into the symmetries of the models using the Lagrangian formalism, distinguishing between continuous and discontinuous symmetries. These symmetries allow for mathematical expediency in the analysis of the model and could also be useful in studying the effect of symmetrical input from distributed cortical regions. Overall, our ability to derive multiple constraints on the fields — and predictions of the model — stems largely from the underlying assumption that the brain operates at a critical state. This assumption, in turn, drives the dynamics towards oscillatory or semi-conservative behaviour. Within this critical state, we can leverage results from the physics literature, which serve as analogues for neural fields, and implicit construct validity. Comparisons between our model predictions and electrophysiological findings from the literature — such as spectral power distribution across frequencies, wave propagation speed, epileptic seizure generation, and pattern formation over the cortical surface — demonstrate a close match. This study underscores the importance of utilizing symmetry preserving PDE formulations for further mechanistic insights into cortical activity.

**Supplementary Information:**

The online version contains supplementary material available at 10.1007/s10827-024-00878-y.

## Introduction

The seminal work by Hodgkin and Huxley in 1954 marked a pivotal moment in neuroscience, introducing the Hodgkin-Huxley model as an initial attempt to capture the intricacies of neuronal dynamics [Hodgkin & Huxley, 1952]. This model, composed of a 4-dimensional, nonlinear ordinary differential equation, delineates the pointwise behaviour of a cell membrane by describing the dynamics of sodium and potassium ions. Through meticulous analysis, it faithfully reproduces the intricate behaviours of voltage-gated ion channels, laying a robust foundation for understanding neuronal membrane dynamics. The Hodgkin-Huxley framework offers flexibility for extension, allowing incorporation of diverse ion-channel types and spatial effects, facilitating modelling of extended membrane phenomena. However, simulating the dynamics of multiple neuronal cells entails solving a complex set of nonlinear differential equations, which poses analytical challenges. To address this, researchers have pursued simplifications, including various neural mass approximations. These simplifications either derive from empirical data, yielding plausible models of neuronal activity, or from simplifying the Hodgkin-Huxley model itself.

Detailed analysis of the Hodgkin-Huxley model revealed that the dynamics of at least two variables need to be included, a variable for membrane potential and a recovery variable. This work resulted in many new simplified models of the neuronal membrane [Catterall et al. 2012, Izhikevich, 2000, Petousakis et al. 2023]. The integrate-and-fire neuron model, or the leaky integrate-and-fire neuron, stands as one of the most straightforward neural mass models. First introduced by Lapicque in 1907, this model predates the seminal work of Hodgkin and Huxley by several decades [Lapicque, 1907, Brunel & Rossum, 2007]. Unlike the intricate Hodgkin-Huxley model, the integrate-and-fire neuron fires a spike once its membrane potential surpasses a certain threshold. These simplified models typically offer a straightforward depiction of the neuronal soma membrane potential. Upon reaching a threshold, they trigger a spike transmission to other neurons, typically inducing synaptic activation in the dendritic tree. These synaptic triggers might manifest elsewhere along the neuron, as explored in the work of Sanchez-Todo et al., which extends to multilevel synaptic triggering in cortical neurons [Sanchez-Todo et al. 2023].

Given the partly stochastic nature of neuronal spiking in the cortex, simplifying the dynamics becomes crucial from a modelling standpoint. Mean field theory, originating from statistical physics, offers a valuable approach where firing rate statistics are analysed across populations of neurons [Deco et al., 2008; Sompolinsky et al., 1988; Treves, 1993; Weiss, 1907]. By employing this framework, one can derive a function that maps the average membrane potential to the average firing rate — typically represented by a sigmoid function — in neuronal populations. The interaction among neuronal populations within a network or sheet is frequently approximated utilizing this sigmoid function. This function effectively maps presynaptic potentials to postsynaptic currents, while a weight-function parameterizes the connection strength between populations [Bressloff & Coombes 2000; Freeman, 1979; Marreiros et al., 2008]. This simplification facilitates the modelling of complex neural networks, providing insights into emergent behaviours and facilitating computational simulations.

Each neuron within a neural network receives inputs from numerous neighbouring neuronal units, collectively contributing to its overall activity. This cumulative effect can be effectively approximated through a summation or integration process. These steps culminate in the derivation of an integro-differential equation that governs the dynamics of individual neurons. Upon further analysis, this integro-differential equation can be explored within the continuum limit, leading to the formulation of neural field equations. The mathematical structures of these fields, along with their experimental validation, have been extensively investigated by pioneering researchers [Amari, 1974; Freeman, 1972; Lopes da Silva et al., 1974; Nunez, 1974; Wilson & Cowan 1972] and further developed by many including [Coombes, 2005; Deco et al., 2008; Destexhe, 1998; Esnaola-Acebes et al., 2017; Jirsa & Haken 1996 & 1997; Liley et al., 2001; Moran et al. 2013; Pinotsis et al., 2012; Robinson et al. 1997; Teleńczuk et al., 2020; Wright & Liley, 1996]. For an in-depth review of neural field models, Cook et al. provide a comprehensive analysis [Cook et al. 2022].

In our investigation, we will delve into the dynamics of neural fields utilizing a partial differential equation (PDE) framework. This formalism enables us to precisely describe a broad range of neural fields, encompassing those amenable to description through the Volterra Kernel method [Cook et al. 2022, Jirsa & Haken, 1997]. By employing this PDE format, we streamline the examination of symmetries inherent in the dynamics. Furthermore, our analysis will closely adhere to the principles of classical field theory, providing a direct physical analogy that enhances our comprehension of the dynamical spectrum of the fields. While our focus primarily revolves around analytical methods or closed-form solutions to the dynamical equations, the PDE formulation offers expediency in numerical analysis. Leveraging the robust tools of PDE modelling facilitates efficient exploration of the dynamics [Laing & Troy, 2003].

Symmetries will play a pivotal role throughout our analysis, and we will consistently make assumptions to preserve these symmetries in subsequent sections. The significance of symmetries in neural fields is underscored by two key features of the brain: self-organized criticality (SOC) and the processing of symmetric information. SOC drives threshold-driven systems towards a critical state between order and disorder, as observed in (Bak et al., 1988; Jensen, 1998) and discussed in (Friston, 2019; Jirsa & Sheheitli, 2022). Accumulating evidence suggests that the brain operates according to the principles of SOC, generating oscillations like those observed in cortical electrophysiological data [Poil et al. 2012; Shew et al. 2009]. Additionally, oscillations are naturally produced in systems exhibiting phase invariance, a key symmetry frequently employed in our study. Through phase invariance, we observe dynamics that approach those of conservative systems; i.e., namely, when non-dissipative, solenoidal dynamics predominate over dissipative gradient flows; e.g., [Friston et al., 2021]. Moreover, empirical evidence suggests that maximizing the processing power of a system occurs when it achieves self-organized criticality (SOC) [Shew et al. 2009]. Finally, there exists a proposition suggesting that regarding the brain as a model for data processing implies enhanced efficiency when the dynamics it operates under align with the symmetries present in the data (Buonomano & Maass, 2009; Ruffini, 2023), leading to generalised synchrony; e.g., [Friston et al., 2021].

Our approach will be structured as follows: In Section 2, we establish a balanced integro-differential equation for a neural field on the cortex, from which we will derive the corresponding partial differential equation (PDE) formulation for cortical fields. We will explore various types of connectivity and their resultant dynamics, encompassing phenomena such as the Klein-Gordon field and limit cycle oscillations. In Section 3, we will introduce the Lagrangian formalism, delving deeper into the symmetries inherent in our model, both continuous and discrete. Moving on to Section 4, we will compare the predictions of the PDE model for cortical fields with experimental evidence. This will include an analysis of the spectral distribution of EEG power across low and high frequencies, examination of seizure onset dynamics, and investigation of pattern formation. Section 5 will serve as a discussion of the findings presented in Sections 2 through 4. We have included an index list for definitions of some of the mathematical terminology used in the paper.

## The cortical field

### Single cortical layer with excitatory and inhibitory neuronal populations

The thickness of the cortex measures only a few millimetres, while its spatial extent spans a much larger scale (approximately 1–2 m^2^, corresponding to the total area of the human cortex). The thinness of the cortical sheet enables us to presume negligible variation in variables across its depth. Our analysis starts by considering two groups of interconnected neuron populations placed in a lattice, thereby creating a two-dimensional surface similar to the cortical surface. We select appropriate definitions of time and length, aimed at minimizing the number of constants in the equations we derive.


1


The cortical sheet is parameterized by **r**, its coordinates. u_e_ and u_i_ represent the activities (postsynaptic dendritic membrane potentials) of excitatory and inhibitory neuronal populations positioned on the cortical sheet. These variables change with location on the cortical sheet, as indicated by their dependence on **r**. The double integral shows that the integral domain covers a surface of the cortical sheet. The function *S* gives the average postsynaptic dendritic membrane potential as a function of the mean firing rate of the targeting neuronal population. w_ee_ is the gain between the incoming activity of a neuronal population and the induced postsynaptic potential. This gain depends on the type of neuronal populations interacting (i.e., excitatory or inhibitory) and the positions on the cortical sheet of both neuronal populations, which is evident from w being a function of two points, **r** and **r**_**0**_. The area element on the cortical sheet is given by $d\Omega$). As formulated, Eq. (1) yield dynamics reminiscent of diffusion or nonlinear wave equations [Cook et al., 2022]. However, with precise balancing, we observe oscillatory dynamics. These oscillations can be succinctly described using a complex field representation. In this framework, the excitatory activity corresponds to the real component of the cortical field, while the inhibitory activity corresponds to the imaginary component. The definition of the complex field is provided by Eq. (2), and its dynamics are governed by Eq. (3). Note that the derivation of oscillatory fields could be achieved without introducing the complex field; however, using a complex field significantly simplifies the derivations, particularly when there is precise balancing between excitatory and inhibitory neuronal populations.

2 $$\varphi ={u}_{e}+i{u}_{i}$$

3

The connectivity (i.e., synaptic gain) between two points, denoted as r and r_0_, is represented by *W* which is a complex number. The connectivity kernel (or sigmoid function) is denoted by *T*, which is a function of the cortical field and its conjugate value. To streamline the equations, we will omit repeated use of evaluation points (e.g., r and r_0_). Note that Eq. (3) is identical to Eq. (1) when precisely balanced for oscillatory activity; however, the dynamical scope of Eq. (3) is less than that of Eq. (1), as the latter allow for both balanced and non-balanced neuronal activity. To derive the partial differential equation (PDE) formulation for cortical field dynamics, we simplify Eq. (3) using the first terms in the Taylor expansion, resulting in a local theory, i.e., a PDE representation. We assume the connection is isotropic, which is why we only include even terms of the Laplacian operator ($\Delta$)4

Additionally, we can differentiate between vertical and horizontal connections along the cortical surface (Eq. 5), which proves valuable in linking neuronal populations across cortical layers. This distinction finds its biological counterpart in the neuronal populations comprising the cortical column, where intralayer connections occur within a cortical column, while intercolumnar connections are deployed horizontally within a cortical layer. A specific example would be when analysing cortical fields corresponding to activity in a brain where each neuronal unit is trapped in a limit cycle, i.e. *The Hopf Whole-Brain Network* [Ponce-Alvarez & Deco, 2024]. The connection in Eq. (5), which leads to limit cycle behavior, comprises two parts. The first term on the right-hand side represents spatially dependent connectivity, while the second term accounts for a very local connection within the cortical microcolumn, modeled by the Dirac delta function ($\delta$) as an approximation of a highly focal connection.

5 $$W\left(r,{r}_{0}\right)T\left(\varphi ,{\varphi }^{*}\right)={W}_{1}\left(r,{r}_{0}\right){T}_{1}\left(\varphi ,{\varphi }^{*}\right)+{W}_{2}{T}_{2}\left(\varphi ,{\varphi }^{*}\right)\delta (r-{r}_{0})$$

Furthermore, we will make the assumption that the gradient of *W* equals 0. However, if there exists directed connectivity along the cortical surface, one would derive a different set of dynamics. A biological analogy for connections with a gradient direction could be in the visual cortex, where distinct directional patterns exist, as evidenced by data analysed in this region [Hubel & Wiesel, 1968]. Nevertheless, we will not delve further into the investigation of dynamics with directional connectivity in this study. The T function, derived from the sigmoid function S in Eq. (1), will be approximated by Eq. (6).

6 $$T\left(\varphi ,{\varphi }^{*}\right)={a}_{1}\left(\varphi +{\varphi }^{*}\right)+{a}_{2}\left(\varphi +{\varphi }^{*}\right)\varphi {\varphi }^{*}+{a}_{3}\left(\varphi +{\varphi }^{*}\right){\left(\varphi {\varphi }^{*}\right)}^{2}$$

The sigmoid function (S) is represented by the (tanh) function, which can be expanded using a Taylor series to yield a summation of odd powers of the argument. Similarly, in the complex setting, the corresponding sigmoid function (T) is also expressed as a Taylor series with odd powers. The dynamics will be analysed using lower order terms from Eq. (4) and Eq. (6). This might be considered too restrictive, and the full analysis has been discussed previously [Jirsa & Haken, 1996 & 1997; Robinson et al. 1997]; however, we will show that even within our framework the dynamics can still be 1) non-trivial and 2) non-linear.

### Lowest order approximation of the field – The Klein Gordon field

We will use the lowest order terms from Eq. (4) and the lowest order term for T (i.e. a_2_ and a_3_ will be 0 in Eq. 6.)7

Introducing some simplifying notations (A and B) we get Eq. (8).

8 $$\frac{\partial }{\partial t}\varphi =-i\varphi +T|_{{{\varvec{r}}}_{0}}A+\Delta T|_{{{\varvec{r}}}_{0}}B$$

9 $$\Delta T={a}_{1}\left({\partial }^{i}{\partial }_{i}\varphi +{\partial }^{i}{\partial }_{i}{\varphi }^{*}\right)$$

We will then have the following dynamics to lowest order (after taking another time derivative).

10 $${\partial }_{t}^{2}\varphi +2iB{a}_{1}{\partial }^{i}{\partial }_{i}\varphi +\varphi +2iA{a}_{1}\varphi =0$$

The above equation is the complex wave equation given that Eqs. (11) and (12) are satisfied.

11 $$2iB{a}_{1}<0$$

12 $$1+2iA{a}_{1}\ge 0$$

Note that both Eqs. (11) and (12) are real. Using terminology from theoretical physics and introducing a speed (c) and mass (m) term we note that the above equation is the complex Klein Gordon (KG) field, Eq. (13).

13 $${\partial }_{t}^{2}\varphi -{c}^{2}{\partial }^{i}{\partial }_{i}\varphi +{m}^{2}{c}^{4}\varphi =0$$

The speed of the waves will be given by *c.* The wave speed (c) is determined by the strength of the connections in the cortical sheet (W) and the steepness of the sigmoid function (T). An increase in connection strength along the cortical sheet results in faster wave propagation, while greater steepness of the sigmoid function at 0 also increases wave speed. The mass term (m) primarily depends on intrinsic (intracolumnar) connections. As local connections within a microcolumn increase relative to connections between microcolumns, the mass term rises, reflecting the energy of the wave contributed by local, non-spatially dependent fields. The dynamics of the cortical field will be approximately given by Eq. (13) where the solution is given by Eq. (14). In Eq. 14, we have introduced the frequency ($\omega$) and the momentum vector (**k**) of the waves. The dispersion equation of the waves will be given by Eq. 15).

14 $$\varphi (\mathrm{r})={\varphi }_{0}{e}^{i\left(\omega t\pm \mathrm{k}.\mathrm{r}\right)}$$

15 $${c}^{2}{k}^{2}+{m}^{2}{c}^{4}={\omega }^{2}$$

### Higher spatial order derivatives of the field

Including the three first terms from Eq. (4) will give us Eq. (16) which we will use to study the effect of higher order spatial derivatives on the dynamics of the cortical fields.16

Using a similar procedure as in Section 2.2, and collecting constant terms and renaming them we get a concise description of the dynamics (Eq. 17). The dispersion relation of the field is given by Eq. (18).

17 $${\partial }_{t}^{2}\varphi -{C}_{1}\Delta \varphi -{C}_{2}\Delta \Delta \varphi +{C}_{0}\varphi =0$$

18 $${C}_{2}{k}^{4}+{C}_{1}{k}^{2}-{\omega }^{2}+{C}_{0}=0$$

### Limit Cycle dynamics

The dynamics of the cortical field will increase as the complexity of the connectivity between cortical units increase. We will here study the dynamics for non-linear *T* where activity gets trapped in a limit cycle. We will also make a distinction between vertical (intracolumnar) and horizontal (intercolumnar) connections using Eq. (5). The connectivity will be defined by Eqs. (19–21). Note that *W*_*2*_ is a constant not dependent on location on the cortical surface.

19 $$W\left({\mathrm{r , r}}_{0}\right)T\left(\varphi ,{\varphi }^{*}\right)={W}_{1}\left({\mathrm{r, r}}_{0}\right){T}_{1}\left(\varphi ,{\varphi }^{*}\right)+{W}_{2}{T}_{2}\left(\varphi ,{\varphi }^{*}\right)\delta ({\mathrm{r - r}}_{0})$$

20 $${T}_{1}\left(\varphi ,{\varphi }^{*}\right)=i{a}_{1}\varphi$$

21 $${T}_{2}\left(\varphi ,{\varphi }^{*}\right)={a}_{3}\varphi +{a}_{4}{\varphi }^{2}{\varphi }^{*}$$

The cortical field dynamics is given by Eq. (3) which together with Eqs. (19–21) will give us the new dynamics with the limit cycle connectivity, Eq. (22).22

Using a similar procedure as in Section 2.2, and after collecting and renaming constant terms we get a concise description of the dynamics (Eq. 23)

23 $${\partial }_{t}^{2}\varphi =-\varphi +{c}_{1}\Delta \varphi -i({c}_{2}\varphi +{c}_{3}\varphi {|\varphi |}^{2})$$

As was stated in the beginning of this section the activity is trapped in a limit cycle which can be seen by analysing the last term of Eq. 23, which is easiest done using polar co-ordinates for the complex field, $\varphi$. These coordinates are defined in Eq. 24.

24 $$\varphi =r{e}^{i\theta }$$

The dynamics in polar coordinates are — after simplification — given by Eq. 25 and 26. Note that we get two expressions as both* r* and $\theta$ are real valued.

25 $$r{\partial }_{t}^{2}\theta +{\partial }_{t}r{\partial }_{t}\theta ={c}_{1}\left(r\Delta \theta +\sum_{i=\mathrm{1,2}}{\partial }_{i}r{\partial }_{i}\theta \right)-({c}_{2}r+{c}_{3}{r}^{3})$$

26 $$-r{\partial }_{t}\theta {\partial }_{t}\theta +{\partial }_{t}^{2}r=-r+{c}_{1}\left(-r\sum_{i=\mathrm{1,2}}{\partial }_{i}\theta {\partial }_{i}\theta +\Delta r\right)$$

If *r* possesses a stable solution, for the bracketed term in Eq. 25, one can derive Eq. 28–29 for the dynamics.

27 $$\sqrt{-\frac{{c}_{2}}{{c}_{3}}}=r$$

28 $$0={\partial }_{t}^{2}\theta -{c}_{1}\Delta \theta$$

29 $$-1={\partial }_{t}\theta {\partial }_{t}\theta -{c}_{1}\sum_{i=\mathrm{1,2}}{\partial }_{i}\theta {\partial }_{i}\theta$$

Equation 28 is the wave equation and Eq. 29 is an inhomogeneous PDE. A particular solution for Eq. 29 is given by Eq. 30 (which also satisfies Eq. 28).

30 $${\theta }_{p}={b}_{1}(t-{t}_{0})+{b}_{2}\left(x-{x}_{0}\right)+{b}_{3}(y-{y}_{0})$$

Equation 28 is solved by the plane wave (${\theta }_{h}$) which will also solve the homogenous part of Eq. 29. Overall, we find a solution for both constraints on $\theta$ (Eq. 28 and 29). A full solution for Eq. 28–29 is given by Eq. 31.

31 $$\theta ={\theta }_{p}+{\theta }_{h}$$

The constrains on the parameters of the solutions are given by Eq. 32.

32 $${b}_{1}=\pm \sqrt{1+{c}_{1}({b}_{2}^{2}+{b}_{3}^{2})}$$

The speed of the wavefront described by the particular solution will be given by Eq. 33.

33 $$\frac{\sqrt{{b}_{2}^{2}+{b}_{3}^{2}}}{\sqrt{1+{c}_{1}({b}_{2}^{2}+{b}_{3}^{2})}}$$

The stability of the limit cycle for weak connections can be noted by examining Eq. 22.3435

The above equation will be stable around the limit cycle provided the spatial derivatives of the cortical field are smaller than the stabilising effect given by the last term. The analysis we present in this section will be valid in the long wavelength approximation.

### Transition between steady state solutions of the field equation.

We aim to examine the transition between semi-stable sets defined by the field equations. Our approach involves simplifying the dynamics by considering the field to be in one of two states at any given point. Specifically, we assume that points in the positive half-plane exhibit behaviour characteristic of a limit cycle, while all other points exhibit activity close to a stable point.

36 $$\left|\varphi \right|\ll 1 \mathrm{if} x<0$$

37 $$\left|\varphi \right|\approx {\varphi }_{l} \mathrm{if} x>0$$

The amplitude of the cortical field during the limit cycle is denoted as ${\varphi }_{l}$. The interconnection among cortical units is described by Eqs. 38–40, like the connectivity outlined in Section 2.3. The *T*_2_ field exhibits a single stable state at 0, encompassed by an unstable limit cycle and a stable limit cycle. In Section 2.4, we previously discussed an unstable stationary state at 0, encircled by a stable limit cycle.

38 $$W\left({\mathrm{r, r}}_{0}\right)T\left(\varphi ,{\varphi }^{*}\right)={W}_{1}\left({\mathrm{r , r}}_{0}\right){T}_{1}\left(\varphi ,{\varphi }^{*}\right)+{W}_{2}{T}_{2}\left(\varphi ,{\varphi }^{*}\right)\delta ({\mathrm{r - r}}_{0})$$

39 $${T}_{2}\left(\varphi ,{\varphi }^{*}\right)={A}_{3}\varphi +{A}_{4}\varphi {\left|\varphi \right|}^{2}+{A}_{5}\varphi {\left|\varphi \right|}^{4}$$

40 $${T}_{2}\left(\varphi ,{\varphi }^{*}\right)={A}_{1}\varphi$$

With the initial conditions (Eq. 36–37) *T*_*2*_ is nearly 0 for all points of the cortex allowing us to approximate the cortical field equation using the Heaviside function ($\Theta$) at the boundary between the 2 regions (Eq. 41).41

Near the boundary in the negative half plane the dynamics is given by Eq. 42.

42 $${\partial }_{t}\varphi =i{a}_{2}{\varphi }_{l}\nabla \varphi$$

The maximum speed of propagation of the front of the limit cycle activity can be estimated using the above equation. Moreover, as the dynamics are invariant to global phase transitions, one can assume that $\varphi$ is a positive real number on the low amplitude activity side at a given time. Maximal front propagation is then estimated as described below.

43 $${\partial }_{t}\left|\varphi \right|={a}_{2}{\varphi }_{l}|\nabla \varphi |$$

The speed of propagation can then be estimated using the following steps.

44 $$\delta t=\frac{\left|{\varphi }_{l}\right|}{{\partial }_{t}\left|\varphi \right|}=\frac{\left|{\varphi }_{l}\right|}{{a}_{2}{\varphi }_{l}\frac{\left|{\varphi }_{l}\right|}{\delta x}}$$

The speed of propagation of the wave front will have a maximal speed given by Eq. 45.

45 $${a}_{2}{\varphi }_{l}=\frac{\delta x}{\delta t}$$

This speed is different from the speed of propagation of waves in the region of low amplitude activity which can be seen on comparing Eq. 8 with 46.

### Multiple cortical layers with excitatory and inhibitory neuronal populations in each layer.

One can now generalise the above treatment to model multiple cortical layers. We will commence with a setup similar to the single cortical layer, but incorporate connections between layers across the cortex and along the cortical surface.46

The *T* function will be given by the following,

47 $$W\left({r}_{0},r\right)T(\varphi ,{\varphi }^{*})={W}_{1}\left({r}_{0},r\right)\varphi +{W}_{2}\varphi \delta (r-{r}_{0})$$

The **W**_**1**_ term is a matrix field and **W**_**2**_ is a matrix. For each point of the cortical surface a matrix is defined interconnecting the cortical layers. Approximating the integral using the Taylor expansion and including constants to simplify the expression we will derive the dynamical equation at the long wave limit.48

Expanding Eq. 48 will give us a wave equation (assuming that |A|< < 1),

49 $${\partial }_{t}^{2}\varphi =-\left(I+{A}_{1}\right)\varphi +{A}_{2}\Delta \varphi$$

Note that the steps between Eqs. 48 and 49 follow the same procedure as in the single-layer case, though these intermediate steps have not been explicitly detailed. If we further assume that the connectivity matrix field only connects neuronal populations within layers and not across layers we get a simplified equation.

50 $${\partial }_{t}^{2}\varphi =-\left(I+{A}_{1}\right)\varphi +{A}_{2}I\Delta \varphi$$

Equation 50 is a linear 2nd order PDE which can be transformed to a system with diagonal matrices (where the components of the ${\mathbf{\varphi }}^{\mathbf{^{\prime}}}$ field are decoupled) if A1 is Hermitian (or symmetric if only real entries), Eq. 51.

51 $${\partial }_{t}^{2}{\varphi }{\prime}-{A}_{2}\Delta {\varphi }{\prime}+D\varphi {\prime}=0$$

The dispersion equation for the following set of equations will be given by Eq. 52.

52 $${\omega }_{i}^{2}={A}_{2}{k}_{i}^{2}-{\mathrm{D}}_{\mathrm{ii}}$$

The distribution of power for the cortical activity for a multilayer system is given by Eq. 53.

53 $$P(k)=\frac{1}{\sum_{i}{A}_{2}{k}_{i}^{2}-{\mathrm{D}}_{\mathrm{ii}}}$$

### Long range connections

Using the above formalism, one can incorporate long-range connections (denoted as **G**) into the cortical field for analysis, enabling analysis of their impact on cortical dynamics. Specifically, our investigation will centre on long-range connections featuring periodic modulation, as outlined by Eq. 54, with n and m serving as integer parameters.

54 $$\mathrm{G}\left(\mathrm{r},\mathrm{r}+{\mathrm{ne}}_{1}+{\mathrm{me}}_{2}\right)=f(n,m)$$

**Fig. 1:**
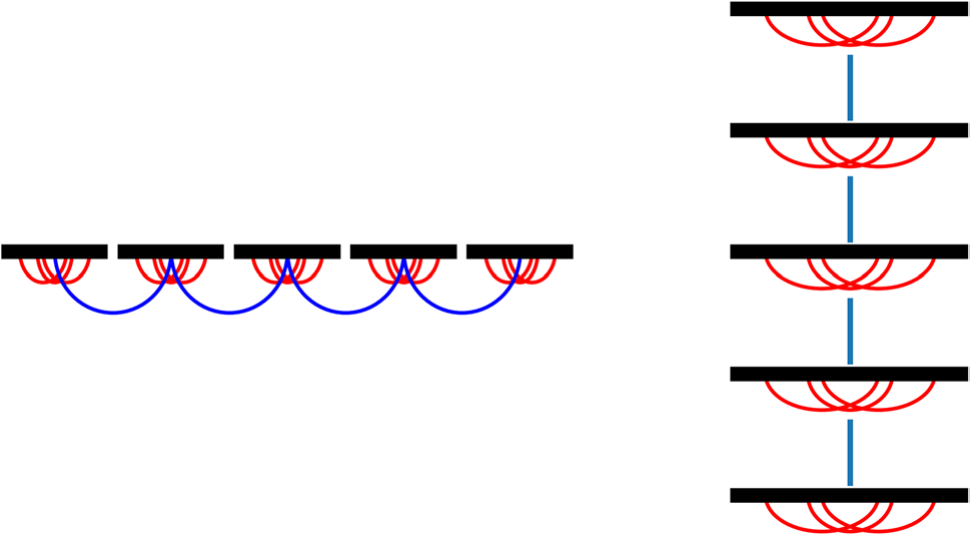
This figure presents two panels depicting the cortical sheet's local and long-range connections. In the left panel, the periodic modulation of long-range connections generates a tessellation of the cortical surface, dividing it into separated sections. Here, local connections interact solely within each tessellation plate. The right panel illustrates the same system with tiles overlaid, facilitating the segregation of local connections into lateral connections and long-range connections into vertical connections, akin to the treatment of connections in Section 2.7. Leveraging this arrangement, we will utilize the identified patterns to estimate self-contained fields

The local connections will mirror those discussed in Section 2.1. Our connectivity arrangement, depicted in Fig. 1, illustrates how the periodic modulation of long-range connectivity will tessellate the cortical surface. Conceptually, the system can be envisioned as tessellations stacked atop one another, with specific connections between them, as illustrated in the right panel of Fig. 1. The system can be segregated into a system with lateral and vertical connections where the local (intrinsic) connections constitute the lateral connections and the long-range (extrinsic) connections the vertical connections, akin to the treatment of connections in Section 2.3. A typical long-range connection might resemble the nearest neighbour connection on a square lattice.

To analyse the dynamics of the cortical field, we will assume that the local dynamics are encapsulated within each lattice square. The dynamics of the entire system are delineated by Eqs. 55–57 (N is the number of lattice squares).

$$F\left(\varphi ,\partial \varphi ,\dots ,{\partial }^{n}\varphi \right) \text{is a linear function on its arguments}$$

55 $$\mathrm F=\mathrm F\otimes\mathrm I$$

56 $$\mathrm{I},\mathrm{ G}\in {\mathbb{C}}^{\mathrm{NxN}}$$

57 $${\partial }_{t}^{2}\varphi =F\left(\varphi ,\partial \varphi ,\dots ,{\partial }^{n}\varphi \right)+G\varphi$$

Assuming that the long-range connectivities exhibit Hermitian or symmetric properties, we can diagonalize the aforementioned equation through a unitary or orthonormal transformation. This process yields a set of decoupled equations, as expressed in Eq. 58, where each lattice tile operates independently from the others.

58 $$\frac\partial{\partial t}\varphi'=F\left(1,\partial^1,\partial^2,\dots,\right)\otimes I\varphi'+D\varphi'$$

The decoupled functions can be restored to their original space by applying the transformation matrix utilised to diagonalise the **G** matrix, as described in Eqs. 59 and 60.

59 $$U{\varphi }{\prime}=\varphi$$

60 $$U({e}_{i}{\prime})={\varphi }_{i}$$

This analysis can be extended to any **G** invariant under a group of transformations, providing various tessellations on the plane. C.f., [Watson et al., 2022].

## The Cortical field and its symmetries

### The Lagrangian formulism

The preceding section's dynamics can be effectively analysed through the Lagrangian formalism. While we'll provide a brief overview here, for a more comprehensive understanding, we recommend referring to the cited sources or any classical fields textbook (Arnol'd IV, 2013; Goldstein et al., 2001; Landau & Lifshitz, 1980). The Lagrangian formalism has been employed since (Lagrange, 1853) to describe primarily conservative dynamics. It uses a variational principle to derive the equations of motion in a relatively straightforward manner, minimizing the need for in-depth knowledge of the dynamical system being analyzed. This formalism facilitates effective analysis of symmetries and conserved quantities within the system. In this section, we provide a brief introduction to how this variational principle can be applied to the dynamics investigated in Section 2

The Lagrangian density is a real-valued function defined over the fields and their derivatives. The action, on the other hand, is obtained by integrating this function across the time-spatial domain occupied by the fields, typically represented as ${\mathbb{R}}^{3}$ or an open/closed subset of it. In this context, such a space corresponds to the cortical surface with a temporal dimension. By extremizing the action, the dynamics of the fields are determined (e.g., via paths of least action). For the dynamics of a cortical field, our focus lies on second-degree derivatives, excluding higher-order derivatives, akin to a long wavelength approximation. The justification for this approach will be explored in Section 4.2. Equation 61 provides the Lagrangian density necessary to capture the dynamics outlined in Section 2.

61 $$L\left(\partial \varphi ,\partial {\varphi }^{*},\varphi ,{\varphi }^{*}\right)={\partial }_{t}{\varphi }^{*}{\partial }_{t}\varphi -{c}^{2}\sum_{i=\mathrm{1,2}}{\partial }_{i}{\varphi }^{*}{\partial }_{i}\varphi -V(\varphi ,{\varphi }^{*})$$

Integrating the Lagrangian density over space–time will give the action.

62 $$S=\int Ld\Omega$$

Extremising this will give the Euler Lagrange field equation as shown in Eq. 63.

63 $${\partial }_{t}^{2}\varphi -\sum_{i=\mathrm{1,2}}{\partial }_{i}^{2}\varphi -\frac{\partial V}{\partial {\varphi }^{*}}=0$$

We introduce the Lagrangian formalism as it aids in grasping the symmetries inherent in the field. Any field transformation that preserves the Lagrangian function will manifest as a symmetry in the dynamics. The Lagrangian density presented earlier exhibits a global U(1) symmetry, indicating that the field can be multiplied by a complex number of unit length without altering the dynamics. This property becomes evident when we rewrite the Lagrangian using a transformed field.

$${\varphi }{\prime}={e}^{i\alpha }\varphi$$

64 $$L\left(\partial \varphi {\prime},\partial {{\varphi }{\prime}}^{*},\varphi {\prime},{\varphi {\prime}}^{*}\right)={\partial }_{t}{e}^{-i\alpha }{\varphi }^{*}{\partial }_{t}{e}^{i\alpha }\varphi -{c}^{2}\sum_{i=\mathrm{1,2}}{\partial }_{i}{{e}^{-i\alpha }\varphi }^{*}{\partial }_{i}{e}^{i\alpha }\varphi -V\left({e}^{i\alpha }\varphi ,{e}^{-i\alpha }{\varphi }^{*}\right)={\partial }_{t}{\varphi }^{*}{\partial }_{t}\varphi -{c}^{2}\sum_{i=\mathrm{1,2}}{\partial }_{i}{\varphi }^{*}{\partial }_{i}\varphi -V\left(\varphi ,{\varphi }^{*}\right)=L\left(\partial \varphi ,\partial {\varphi }^{*},\varphi ,{\varphi }^{*}\right)$$

### Multilayer Cortex

Turning now to the multilayer formulation, the Lagrangian density governing the dynamical equations for the multilayer cortex, derived in Section 2.3, is represented by Eq. 65.

65 $$L\left(\partial \varphi ,\partial {\varphi }^{*},\varphi ,{\varphi }^{*}\right)={\partial }_{t}{\varphi }^{*}{\partial }_{t}\varphi -{c}^{2}\sum_{i=\mathrm{1,2}}{\partial }_{i}{\varphi }^{*}{\partial }_{i}\varphi -{m}^{2}{\varphi }^{*}\varphi$$

Moreover, this Lagrangian maintains invariance under unitary inversions, a property evident when transforming the density as depicted in Eq. 66.

66 $$L\left(\partial \left(U\varphi \right),\partial \left({\varphi }^{*}{U}^{*}\right),U\varphi ,{\varphi }^{*}{U}^{*}\right)={\partial }_{t}{\varphi }^{*}{U}^{*}{\partial }_{t}U\varphi -{c}^{2}\sum_{i=\mathrm{1,2}}{\partial }_{i}{\varphi }^{*}{U}^{*}{\partial }_{i}U\varphi -{m}^{2}{\varphi }^{*}{U}^{*}U\varphi =L\left(\partial \varphi ,\partial {\varphi }^{*},U\varphi ,{\varphi }^{*}\right)$$

We have demonstrated that a set of non-interacting layers exhibits a global U(N) symmetry, which becomes broken upon introducing interactions between the layers. The Lagrangian for interacting fields is represented by the following expression, where **A** is a matrix. In Section 2.2, we illustrated that a linear transformation of the fields is possible (provided **A** is Hermitian, symmetric regarding the real components, and antisymmetric regarding the imaginary components) that decouples the equations. However, it's important to note that the Lagrangian also undergoes transformation under this linear operation.

67 $$L\left(\partial \varphi ,\partial {\varphi }^{*},\varphi ,{\varphi }^{*}\right)={\partial }_{t}{\varphi }^{*}{\partial }_{t}\varphi -{c}^{2}\sum_{i=\mathrm{1,2}}{\partial }_{i}{\varphi }^{*}{\partial }_{i}\varphi -{m}^{2}{\varphi }^{*}A\varphi$$

### Long range connections

In Section 2.3 we investigated long distance connections with a periodic modulation. The lagrangian function will be given by Eq. 68.

68 $$L\left(\partial \varphi ,\partial {\varphi }^{*},\varphi ,{\varphi }^{*}\right)=\sum_{a}\left[{\partial }_{t}{\varphi }_{a}^{*}{\partial }_{t}{\varphi }_{a}-{c}^{2}\sum_{i=\mathrm{1,2}}{\partial }_{i}{\varphi }_{a}^{*}{\partial }_{i}{\varphi }_{a}-V\left({\varphi }_{a},{\varphi }_{a}^{*}\right)\right]+{\varphi }^{*}G\varphi$$

The G matrix delineates the long-range connectivity, ensuring that the connectivity field possesses a discrete symmetry. This distinct symmetry stands in contrast to the continuous symmetries discussed earlier. For discrete symmetries within the Euclidean plane, we can enumerate them where n and m are integers, Eq. 69.

69 $$G\left(r,r+n{e}_{1}+m{e}_{2}\right)=f(n,m)$$

This concludes our formal set up. In the next section, we use the functional forms above to ask what kind of dynamics were would predict under this model, and whether such dynamics could be characterised as cortical in nature.

## The Cortical field model: Predictions and experimental results

In this section, we undertake a comparative analysis between the predictions generated by the cortical field model and empirical data. Section 4.1 replicates the analytical approach employed by Nunez in his examination of the *Brain Wave Equation*, comparing model predictions using the cortical field presented in this study with electrophysiological observations from the human brain [Nunez, 1974]. Moving forward, Section 4.2 explores the spectral properties inherent in the cortical field, elucidating the characteristic inverse polynomial decay and contrasting it with experimental findings. Sections 4.3 and 4.4 delve into the dynamics of limit cycles and the impact of long-range connections on the cortical field, respectively, providing a comprehensive comparison to experimental data.

### Propagation speed of large wavelength fields

In the previous section, we established a partial differential equation (PDE) formulation for the cortical field. In the long wavelength approximation, we obtain the Klein-Gordon (KG) field equation with two key parameters: the speed of wave propagation and a mass term. Experimental investigations have identified traveling waves in cortical tissue, exhibiting speeds ranging from 0.001 to 30 m/s (Bragin et al., 1999; Chervin et al., 1988; Dalva et al., 1997; Domich et al., 1986; Hall & Kuhlmann, 2013; Muller et al., 2018 & 2018; Sanchez-Vives & McCormick, 2000; Staba et al., 2002; Wester & Contreras, 2012; Xu et al., 2007). The dynamical equations (see Eq. 7) will yield an expression for the speed, as defined in Eq. 70.70

Certain parameters in Eq. 63 are subject to geometric constraints, such as the area over which connectivity integration is required. We will assume this area to be significantly larger in magnitude than the size of a cortical column or hypercolumn, typically with a radius approximately equal to 1 mm (Dalva et al., 1997; Mountcastle et al., 1955). Consequently, we can estimate the integral terms in Eq. 70.7172

Additionally, we will make the assumption that W is non-zero within a region roughly equivalent in size to a hypercolumn. This assumption leads us to establish order of magnitude relations for W and its derivatives

73 $$W\sim \Delta W\sim \Delta \Delta W$$

Empirical studies have estimated the speed of propagation to fall within the range of 1–1000 mm/s. Another parameter that can be estimated from electrophysiological investigations of the human brain is the frequency of oscillation of neuronal populations.

74 $${a}_{0}\sim {10}^{1}-{10}^{3} {\mathrm{rads}}^{-1}$$

Utilizing Eqs. 70–74, we can derive a constraint on the speed of the waves and the connectivity, assuming that Eq. 70 is predominantly influenced by the *a*_*0*_ term.

75 $$\begin{array}{c}1>\frac{{A}_{1}W}{{a}_{0}}\sim \frac{{c}^{2}}{{a}_{0}^{2}}\\ c<{a}_{0}\sim {10}^{1}-{10}^{3}\\ {A}_{1}W<{10}^{1}-{10}^{3}\end{array}$$

The connectivity term exhibits a broad range of variation, reflecting the varying speeds and oscillatory activities observed in experimental data across different frequencies. Similarly, the mass term parameterising the Klein-Gordon (KG) field can also be estimated. The estimated range (Eq. 76) indicates that it does not play an important role in the dynamics for frequencies noted in electrophysiological recordings (< 1 Hz). This fact is further supported by data as the one over frequency squared distribution is seen down to at least 0.1-1 Hz.

76 $$m\sim \frac{{a}_{0}}{{c}^{2}}\sim {10}^{-3}-{10}^{0}$$

### Higher order differential terms in the dynamical equations

In Section 2.1.2 we derived the cortical field upto 4th order spatial derivatives. The dynamical equation will be given by Eq. 77.77

Renaming and introducing new constants will allow us to rewrite Eq. 77.

78 $${\partial }_{t}^{2}\varphi =-{a}_{0}^{2}\varphi -{a}_{1}\varphi +{a}_{2}\Delta \varphi -{a}_{3}\Delta \Delta \varphi$$

The dispersion relation (Eq. 79) derived from Eq. 78 can be used to estimate the power distribution over frequency for the cortical field.

79 $${\omega }^{2}={a}_{0}^{2}+{a}_{1}+{a}_{2}{\mathrm{k}}^{2}+{a}_{3}{\mathrm{k}}^{4}$$

80 $$P\left(f\right)=\frac{1}{{a}_{0}^{2}+{a}_{1}+\frac{{a}_{2}}{{c}^{2}}{\mathrm{f}}^{2}+\frac{{a}_{3}}{{c}^{4}}{\mathrm{f}}^{4}}$$

**Fig. 2:**
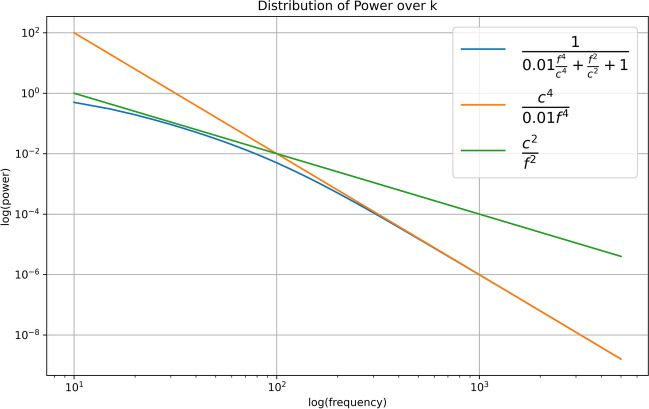
The figure illustrates the power distribution across frequency within the cortical field. A gradient of -2 is observed in the lower frequency band, contrasting with a steeper gradient of -4 in the higher frequency bandwidth. Notably, when a velocity (c) of 10mms^−1^ is applied, the frequency threshold at which the gradient shifts occur at 100 Hz

Comparing with power distributions for electrophysiological data we have a one over frequency squared distribution between 1-100 Hz. At the upper end of this range there is a further reduction in power with a one over frequency to the power 4 [Miller et al. 2009]. Figure 2 illustrates the spectral distribution for the modelled equation (Eq. 80), with a shift in gradient at around 100 Hz [Miller et al. 2009]. A propagation speed of 10mms^−1^ was utilized in Fig. 2. Notably, the ratio between the f^4^ and f^2^ -term in the denominator is 100 in the figure, highlighting that the change in gradient is attributed to the speed of wave propagation (c in Eq. 80).

The linear Klein–Gordon equation can be expanded using higher-order terms from the Taylor expansions in Eqs. 4 and 6. In this section, we expanded using higher-order terms from Eq. 4, specifically second and quartic spatial derivatives. Higher-order terms from the sigmoid function expansion (Eq. 6) can also be included. Incorporating higher-order terms from both the derivative expansion (Eq. 4) and the sigmoid function will similarly affect the dispersion equation.

81 $${\omega }^{2}={a}_{0}^{2}+{a}_{1}+{a}_{2}{\mathrm{k}}^{2}+{a}_{3}{\mathrm{k}}^{4}+{b}_{1}+{b}_{2}{\mathrm{k}}^{2}+{b}_{3}{\mathrm{k}}^{4}$$

The a-coefficients are derived from the spatial derivative terms, and the b terms from the non-linear sigmoid function. Similar to Eq. 79, we have a non-constant gradient for the power-over-frequency graph. The change in the gradient of the dispersion equation seen in experimental data is likely due to a combination of higher spatial derivatives and the non-linearity of the sigmoid function. Previous estimates of spectra from neural field theories have shown similar frequency dependent gradients as discussed above [Evertz et al., 2022; Rennie et al., 2002; Wright & Liley, 1996].

### Limit cycle dynamics

In Section 2.3 the dynamics of oscillators trapped in limit cycles were estimated where the phase of the solution is given by the sum of two wave equations, Eq. 82 and 83.

82 $${\theta }_{p}={b}_{1}(t-{t}_{0})+{b}_{2}\left(x-{x}_{0}\right)+{b}_{3}(y-{y}_{0})$$

83 $${\theta }_{h}={A}_{0}{e}^{i(\omega t-k.r)}$$

The activity of the dynamics will then be given by the following expression.

84 $$\varphi ={e}^{i\left({\theta }_{p}+\sum {\theta }_{h}\right)}$$

**Fig. 3:**
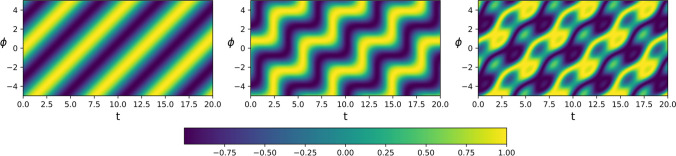
This figure illustrates the cortical field generated around the stable state of limit cycle oscillations. These oscillations adhere to the wave equation, with distinct frequency waves depicted in the left and middle panels, and a mixture of waves shown in the right panel. The amplitude of the cortical field is given in arbitrary units according to the colour map

Figure 3 show different types of field solutions.

The propagation of a high amplitude wavefront can be modelled using the above results. The dynamics at the boundary between a region with high and a low amplitude activity will be investigated. We provide a qualitative analysis of the propagation of high amplitude activity and compare the ensuing theoretical predictions with experimental results from the literature. More specifically, we will investigate a region with high amplitude activity (region A) placed along the negative x-axis with low amplitude activity along the positive x-axis (region B). The dynamics at the boundary between high and low amplitude activity is given by Eq. 85 and the dynamics of the phase in region A is given by Eqs. (86) and (87) (we have re-defined some constants in Eq. 28, 29 and 42 to shorten the notation).

85 $${\partial }_{t}\varphi =\frac{i}{2}\nabla \varphi$$

86 $${\partial }_{t}^{2}\theta =\frac{1}{2}\Delta \theta$$

87 $$1={\partial }_{t}\theta {\partial }_{t}\theta -\frac{1}{2}{\partial }_{x}\theta {\partial }_{x}\theta$$

A particular solution for Eq (87) is given by Eq. (88).

88 $${\theta }_{p}=\sqrt{2}\left(t+x\right)$$

As noted in Eq. (85) the rate of change of the cortical field comes with a phase change of 0.5 $\pi$ and we will assume that the activity in region B (low amplitude activity) will transition to high amplitude activity with the same phase shift in relation to the high amplitude activity of regions A.

89 $${\varphi }_{0+}\to -i{\varphi }_{0-}$$

This will cause a shift of phase of -i at the boundary between high and low amplitude activity. We will investigate how this discontinuity will affect the dynamics by modelling the phase shift as an initial condition using the Heaviside function.

90 $${\theta }_{IC}(x)=\pi \left(\frac{1}{2}- \Theta \right)$$

The time dependent solution of the dynamics (Eq. (86)) is given in Eq. (91) (tilde indicates the Fourier transform) together with the dispersion relation for the individual waves (Eq. (92)).

91 $$\theta \left(t,x\right)={\int }_{-|{k}_{m}|}^{|{k}_{m}|}{\widetilde{\theta }}_{IC}\left(k\right){e}^{i(kx\pm \omega t)}dk$$

92 $$\omega =\pm \frac{k}{\sqrt{2}}$$

Combining both equations and using results from Fourier analysis we can estimate the time dependent phase (Eq. (93)).

93 $$\begin{array}{c}\theta \left(t,x\right)={\int }_{-|{k}_{m}|}^{|{k}_{m}|}{\widetilde{\theta }}_{IC}\left(k\right){e}^{i(kx\pm \frac{k}{\sqrt{2}}t)}dk\\ \theta \left(x\pm \frac{1}{\sqrt{2}}t\right)={\int }_{-|{k}_{m}|}^{|{k}_{m}|}{\widetilde{\theta }}_{IC}\left(k\right){e}^{ik(x\pm \frac{1}{\sqrt{2}}t)}dk={\int }_{-\infty }^{\infty }{\widetilde{\theta }}_{IC}\left(k\right){e}^{ik\left(x\pm \frac{1}{\sqrt{2}}t\right)}rect(-{k}_{m},{k}_{m})dk\\ \theta \left(x\pm \frac{1}{\sqrt{2}}t\right)={\theta }_{IC}\left(x\pm \frac{1}{\sqrt{2}}t\right)\otimes sinc\left({k}_{m}\left(x\pm \frac{1}{\sqrt{2}}t\right)\right)\end{array}$$

Equation (84) provides a qualitative estimation of the transition between low and high amplitude activity. The phase function derived in Eq. (93) diminishes with both time and distance and to model this transition more precisely, we will utilize Eq. (94).

94 $$\varphi ={r}_{0}{e}^{i\theta }+r(t){e}^{i{a}_{0}t}$$

**Fig. 4:**
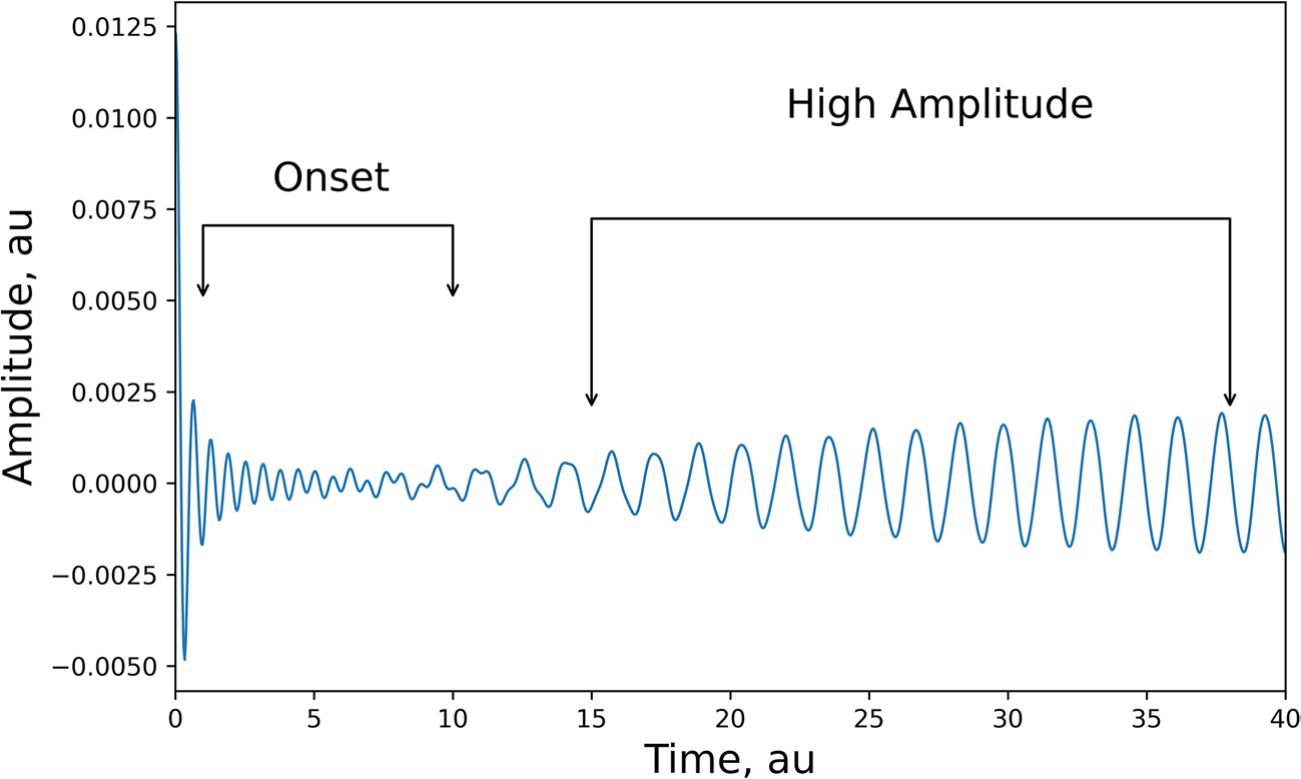
This figure illustrates the transition from low to high amplitude activity. While the time scale is arbitrary, a rapid onset of low amplitude activity is observed, followed by slower oscillations of higher amplitude. This pattern bears resemblance to the stereotypical electrographic seizure onset pattern

We introduced *r(t)* as a monotonically increasing function ranging from 0 to a predetermined value, which ultimately dominates the oscillatory behaviour in the limit cycle phase. In Fig. 4, we illustrate Eq. (94) graphically, depicting the transitional shift from low to high amplitude activity. Notably, the onset exhibits rapid initial activity before the dynamics stabilize into the steady state of the limit cycle. Crucially, this phenomenon parallels observations in experimental studies of human brain activity during transitions from spontaneous to seizure states, where a similar pattern emerges: fast low-amplitude onset preceding pronounced rhythmic firing of higher amplitude—a pattern often recognized as the stereotypical onset of epileptic seizures [Jirsa et al., 2014; Kramer et al., 2007; Lagarde et al. 2019].

Recent empirical work on seizure onset has shown a multitude of different seizure onset patterns and also off set patterns which have been elegantly modelled using bifuctiona analysis of an underlying neuronal model [Saggio et al. 2020]. These bifurcations can be analysed using the given framework of the presented model and will occur as the connection strength increases in gain. The stability of the system near zero will be lost and there will be a new locus of high amplitude stable states. As the system changes between these stable states as the gain in the sigmoid function changes several of the observed and modelled seizreu patterns could be described usning the cortical field model framework presented in this paper.

### Long range connectivity

In Sections 2.7 and 3.4, we examined the impact of long-range connectivity on the dynamics of cortical fields. Our focus was on exploring connectivity strengths characterized by a periodic recurrence, leading to discrete symmetries within the cortical field. Here, we aim to further investigate the various types of activity that long-range connectivity, periodically modulated, will induce. These dynamics are encapsulated by Eq. (95).

95 $${\partial }_{t}^{2}\varphi =L\left(1,{\partial }^{1},{\partial }^{2},\dots ,\right)I\varphi +G\varphi$$

The first term of Eq. (95) encapsulates the local connections, while the second term represents the contributions from long-range connections. In Section 2.7, we explored self-contained fields characterized by periodic modulations generated by the symmetries of the G matrix. The solutions to Eq. (95) are provided by Eq. 60, where **e**_**i**_**’** denotes solutions to the diagonalized system, which in turn satisfy Eq. (96).

96 $${\partial }_{t}^{2}{e}_{i}{\prime}=L\left(1,{\partial }^{1},{\partial }^{2},\dots ,\right)I{e}_{i}{\prime}+{U}^{*}GU{e}_{i}{\prime}$$

**Fig. 5:**
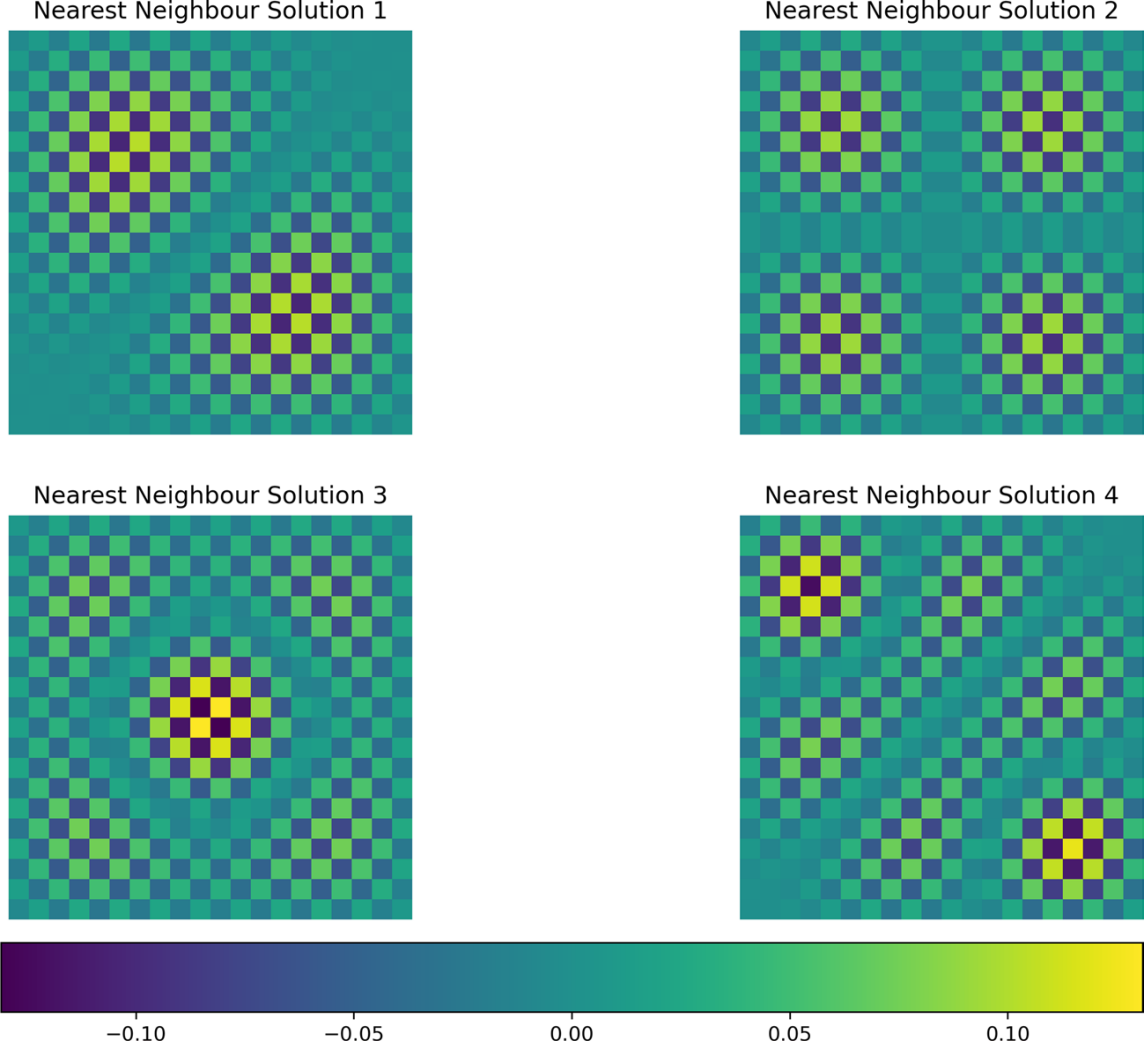
This figure depicts self-contained activity within a system characterized by nearest neighbor connectivity. This system encompasses 625 distinct self-contained patterns, with four of these patterns showcased in the figure. Each square within the figure represents a solution to the diagonalized system, scaled by a constant factor indicated on each tessellation plate. The value of the constant factor is given by the colour map

**Fig. 6:**
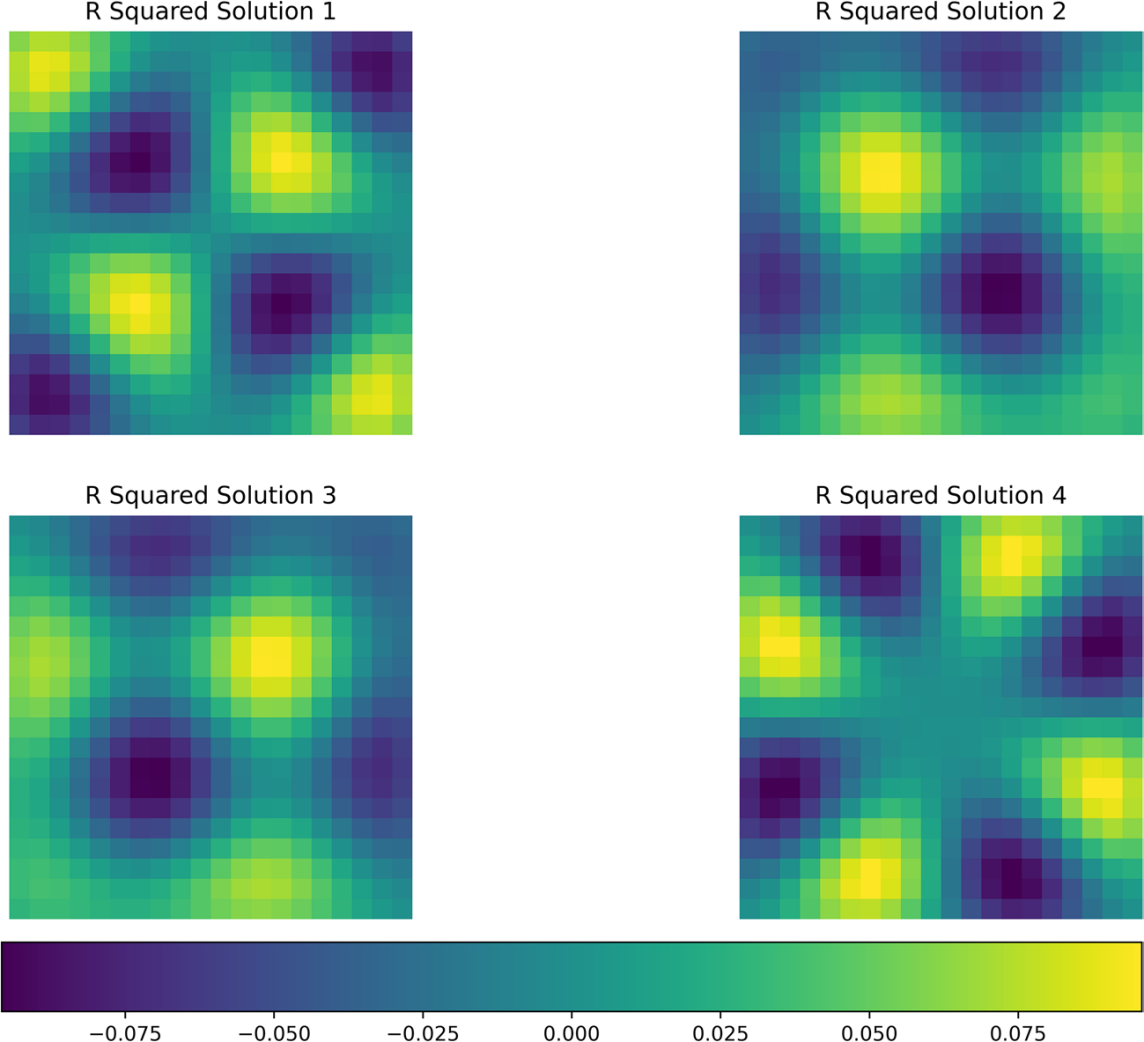
The figure depicts self-contained activity within a system characterized by squared distance connectivity. This system encompasses 625 distinct self-contained patterns, with four of these patterns showcased in the figure. Each square within the figure represents a solution to the diagonalized system, scaled by a constant factor indicated on each tessellation plate. The value of the constant factor is given by the colour map

We can demonstrate this through examples. For instance, a nearest-neighbour G-connectivity scheme among square patches of the cortical surface will result in the self-contained fields depicted in Fig. 5. Similarly, G-connectivity with connection strengths inversely proportional to the distance between patches will yield the self-contained field illustrated in Fig. 6. We now turn to a discussion of these results in light of empirical phenomena and related models of those phenomena.

It should be noted that the dynamics used for the local connections are Lagrangian, not necessarily linear. The derivations and simulations in Section 4.4 demonstrate the spatial dynamics for any Lagrangian dynamic under the influence of long-range connections. As noted in Section 4.2, higher expansion terms of the sigmoid function introduce non-linear terms in the neural field equations of motion. The non-linear wave equation has been shown to generate both soliton and steady-state wave (or bump) solutions, yielding patterned results similar to those shown in [Laing & Troy, 2003].

## Discussion

In this study, we offer a mathematical analysis of the dynamics of neural or cortical fields, articulated through partial differential equations (PDEs). Our exploration encompasses a spectrum of activities, ranging from single-layer to multi-layer configurations, and encompasses both local and long-range connections. Specifically, we delve into the dynamics stemming from limit cycle behaviour in cortical units, investigating phenomena such as the propagation of states from low to high amplitude. Leveraging the PDE formalism enables us to probe the symmetries inherent in the dynamics, particularly evident when utilizing the Lagrangian formalism. By expressing the dynamics in this manner, we uncover symmetrical patterns that may underlie the complex behaviour of cortical fields. To validate our theoretical framework, we juxtapose our predictions with electrophysiological data from the brain, providing both quantitative and qualitative assessments. This study not only furnishes empirical grounding for the models discussed but also establishes connections with the extensive knowledge base of dynamical and Lagrangian systems in theoretical physics.

In Section 4.2, we delineated the spectral distribution characterizing cortical dynamics. The consistent finding of an approximate -2 gradient in the log–log spectral distribution across multiple investigations underscores its robustness and prevalence in electrophysiological studies of the human brain [Jirsa 2009]. This phenomenon primarily stems from fundamental terms in the PDE governing cortical models, such as the wave equation, which assumes a long wavelength approximation; effectively minimizing the influence of higher spatial derivatives. Miller et al. demonstrated this -2 gradient for frequencies below 70-80 Hz, transitioning to a -4 gradient at higher frequencies, aligning with the behaviour anticipated by the PDE governing cortical activity [Miller et al., 2009]. Notably, this experimental observation demarcates the frequency range where the wave equation remains valid, encompassing the primary frequency bands of interest in clinical electrophysiology (1-40 Hz) (Schomer & Lopes da Silva, 2017). Given that empirical data exclusively reveals -2 and -4 gradients in the log–log spectral distribution, it is improbable that higher-order spatial derivatives significantly contribute to the dynamical equations. Furthermore, the granularity of the cortical surface imposes an upper spatial frequency limit on activity, with wavelengths ranging approximately from 0.1-1 mm, corresponding to the dimensions of the smallest neuronal unit, assumed to be that of a cortical column. The frequency content and distribution of electrographic activity have been consistently measured using both non-invasive and invasive methods in humans, where the main spectral content shows broad band activity, approximately 1-200 Hz (Schomer & Lopes da Silva, 2017). The frequency bandwidth falls within the range of the theoretical predictions of the neural field model, where the spatial extent and its graininess determine the upper and lower bandwidth limits. The lower limit, as mentioned previously, is the size of a cortical column, with an upper limit of approximately 0.1-1 mm. There does not seem to be a consensus in the literature on the reason for the inverse relationship with frequency in electrographic data, but several theories have been presented including phase stability, critical states, and biophysical filtering [Bedard et al. 2006, Buzsaki et al. 2009; Thatcher et al., 2009]. While our study demonstrates that the overall connection over neuronal populations affects cortical activity dynamics, it should be noted that our model fails on scales smaller than that of a cortical column. However, it is reasonable to expect that the spatial filtering of dendrites continues to play a role in neuronal activity on smaller scales.

In Sections 2.3 and 4.4, we delved into the cortical activity arising from neuronal populations confined within limit cycles, a subject extensively explored in existing literature. The Hopf transition model has been a frequent point of reference in these investigations. Recently, there has been a focus on exploring activity among a network of Hopf models within the broader context of the entire brain, particularly around the stable zero point [Ponce-Alvarez & Deco, 2024]. Here, we examine a refined variant of the Hopf model, encompassing a blend of spatially dependent and independent connectivity among cortical units. By "spatial," we refer to the two-dimensional space of the cortical sheet, where the connectivity inducing limit cycle activity traverses the surface within the cortical sheet. The system manifests several stable states, notably the null state (with zero activity) and a collective limit cycle state involving all oscillators (referred to as the limit-cycle state). Perturbations around the limit cycle state unveil wave equations, akin to real Klein-Gordon field equations, governing the oscillator phase. Notably, the propagation speed of these waves differs from that observed in the stable zero state.

Furthermore, we scrutinized the transition of activity between the zero state and the limit cycle state, a study previously undertaken for finite networks [Cooray et al., 2023 and 2024]. Leveraging qualitative arguments, we infer a plausible shift in cortical activity from a state of minimal activity to one of high amplitude, similar to earlier computational work [Kramer et al., 2007; Jirsa et al. 2014]. Strikingly, this transition bears resemblance to electrophysiological data recorded during seizure onset in the human brain [Lagarde et al. 2019]. Such findings bolster the suitability of partial differential equation (PDE) descriptions for understanding cortical waves and the changes observed in experimental recordings. Recent empirical work on seizure onset has revealed a multitude of different onset and offset patterns, which have been elegantly modeled using bifurcation analysis of an underlying neuronal model [Saggio et al., 2020]. These bifurcations can also be analyzed using the framework presented in this paper. The linear approximation analyzed in Section 2 was constrained by weak connection strength between neuronal units. If the connection gain increases, the non-linearity of the sigmoid function will lead to an instability of the stable state at 0, resulting in a bifurcation with an unstable zero point surrounded by a locus of stable points. The spatiotemporal dynamics are intricate; however, similar to the neural mass study in [Saggio et al., 2020], we will observe similar changes in dynamics. The PDE formulation, as opposed to the ODE (or neural mass) formulation, is useful when considering the effect of spatiotemporal dynamics and not just temporal dynamics. This type of data is still not widely available in patient studies, such as invasive recordings to identify the seizure onset zone. However, the framework presented here could serve as a starting point for predicting changes that might be observed in ultra-high-density recordings of seizures.

It is worth noting that the model we employ, featuring limit cycle oscillators, diverges from the Kuramoto model, which permits robust connectivity between spatially separated cortical regions. In contrast, our model is local, providing a PDE-based depiction of cortical activity. For an in-depth review of the Kuramoto model, see [Breakspear et al. 2010], where patterns of cortical activity are derived by introducing periodic components into the connectivity field. We adopt a similar methodology to explore long-distance connections in contrast to local connections, particularly focusing on activity around the zero state.

In Sections 2.7 and 4.5, we delve into the impact of both long-distance and local connections on the dynamics of the cortical field. As previously explored by numerous authors, we introduce periodically modulated connections between neuronal or cortical units. The mathematical framework we employ for these models facilitates the efficient derivation of these fields, thereby enabling a clear understanding of how these long-range connections influence cortical activity. The model under scrutiny can be conceptualized as a tiled (i.e., tessellated) cortical field, with each patch of the cortical surface within the periodic connections resembling a tile connected to other tiles, see Fig. 1. The long-range periodically modulated connections introduce symmetries in the tile-to-tile connections, thereby resulting in discrete symmetries of the cortical field if studied without tiling the cortex, a method we utilize to derive the potential patterns of the fields. Leveraging the discrete symmetry induced by the long-range connectivity, we derive a series of potential patterns of cortical activity akin to those estimated in computational analyses of neural fields and observed in empirical data [Amari, 1977; Atay & Hutt, 2006; Bressloff & Cowan, 2002; Bressloff, 2003; Bosking et al., 1997; Coombes & Owen, 2005; Ermentrout & Cowan, 1979; Hutt et al., 2003; Laing, 2005; Moser et al. 2008]. The multi-tiled cortical surface represents a spatial hierarchical model of the cortical surface, facilitating a model of parallel processing of data within the cortex. Notably, patterns of connections and cortical activity have been observed in various brain regions, suggesting that the utilization of parallel processing of data could be advantageous, particularly in regions such as the hippocampus and visual cortex [Hubel & Wiesel, 1968; Lee et al. 2020; Moser et al. 2008].

Throughout this paper, we pay special attention to the symmetries of the dynamical models. These have been introduced partly without clear biological justification; however, the dynamics revealed share many characteristics with those seen in electrophysiological data from the brain. In summary, we employ the following symmetries: unitary symmetries within each cortical column and discrete symmetries for long-range connections. The unitary invariance between excitatory and inhibitory units allows for the generation of oscillatory states, which are ubiquitously observed in the brain. From a mathematical perspective, it allows for the phase space of cortical dynamics to be symplectomorphic (with a Hamiltonian system), at least approximately within a time frame. We further extend this assumption to the connections between different cortical layers, assuming them to be Hermitian, allowing the dynamics again to be sustained, albeit approximately only within a time frame. Breaking these symmetries (e.g., by having connectivity that does not respect the symmetry) would result in quickly attenuating activity. This might be the case in the actual cortex; however, one would then be left with the cortical activity resulting from the remaining connectivity fields that are Hermitian, allowing the work presented in this study to be implemented on the smaller subspace of activity which is semi-conservative. Finally, we assume discrete symmetries in the long-range connection, which result in a tessellated pattern of cortical activity. The patterned activity has been shown in several experimental studies of cortical surfaces in both hippocampal and visual cortex but also in several other regions of the brain [Hubel & Wiesel, 1968; Lee et al. 2020; Moser et al. 2008]. Moreover, there is increasing evidence that cortical structures inherit several symmetries from nature, where it has been suggested that symmetric data is efficiently processed when the model inherently has the same symmetries (Miao & Rao, 2007; Ruffini, 2017 and 2023). This can be read in terms of the good regulator theorem [Conant & Ross 1970] or modern day treatments in which the brain entails a generative model of the world in which it is immersed: e.g., [Friston et al., 2012; Friston et al., 2021].

In conclusion, this study derives a PDE formulation of cortical fields estimated in a brain dominated by oscillations. To maintain the oscillatory behaviour of the model, we had to maintain the underlying symmetries of the model, which have been included previously but foregrounded in the current work. The main predictions of the model fall within the realm of experimental findings and are similar to other neural field models. In contrast to numerical characterizations of neural field models, we have focused on a highly symmetric model allowing us to define it using the Lagrangian technique used in theoretical field study (from theoretical physics). The assumptions and approximations performed in the derivations could be criticized for being overly simplistic and constrained. However, the goal was to present a field model retaining some of the important characteristics of neural fields yet being simple enough for analytic investigation. Crucially, the ensuing cortical field shows symmetries similar to that seen in electrophysiological data from the brain and also in the data the brain itself models. Overall, our ability to derive multiple constraints on the fields and predictions of the model stems largely from the underlying assumption that the brain operates at a critical state. This assumption, in turn, drives the dynamics towards oscillatory or semi-conservative behaviour. Within this critical state, we can leverage results from the physics literature, which serve as analogues for neural fields, facilitating our understanding of cortical dynamics. Further elaboration of the cortical field as discussed above, including information coding and processing properties, could be of interest within the field of cognitive neuronal computation.

## Supplementary Information

Below is the link to the electronic supplementary material.

Supplementary file1 (DOCX 16 KB)

## Data Availability

Not applicable.
