# Supplementary material for: A cortical field theory – dynamics and symmetries
Source: J Comput Neurosci. 2024 Oct 1;52(4):267–84. doi: 10.1007/s10827-024-00878-y (PMC11470901; doi:10.1007/s10827-024-00878-y)
Supplement: Supplementary file 1 — Supplementary file1 (DOCX 16 KB) [file 10827_2024_878_MOESM1_ESM.docx]

Index list

Scalar variables will be written in italics

Vector or Matrix valued variables will be written in bold.

z/**z** is a complex variable/vector

z* is the complex conjugate

u_e/I_ is a real valued cortical field from excitatory/inhibitory neuronal units.

a, b, c, d, A and B are used for constant coefficients and are either real or complex valued.

W is a scalar weight function

**W** is a matrix of weight functions

**G** is used for a weight matrix with discrete symmetry

**I** is the identity matrix

**U** is a unitary matrix (i.e. **U*U**=**I**)

**U*** is the conjugate transpose of the matrix **U**

T is a scalar function mapping activity between different regions of the cortex

∆ is the Laplacian operator

$\delta t$ will indicate a small change in the variable t

$\nabla$ is the gradient operator

$\frac{\partial}{\partial x_{j}}=\partial_{j}$ is the partial differential in the direction of the jth co-ordinate direction

Repeated indices are summed over e.g.

$$\partial^{i}\partial_{i}=\sum_{i=1,2} \partial_{i}\partial_{i}$$

$\varphi\mathbb{\in C}$ is a scalar cortical field

$\boldsymbol{\varphi}\in\mathbb{C}^{n}$ is a vector cortical field with n components

$\theta\mathbb{\in R}$ is the phase of the cortical field

$\chi\mathbb{\in R}$ is a cortical field

$\omega\mathbb{\in R}$ is 2π times the frequency of a wave solution

$\mathbf{k}\in\mathbb{R}^{2}$ is the momentum vector of a wave solution

*L* is used for the Lagrangian density
